# Leadership in the clinical workplace: what residents report to observe and supervisors report to display: an exploratory questionnaire study

**DOI:** 10.1186/s12909-015-0480-5

**Published:** 2015-11-02

**Authors:** Martha A. van der Wal, Fedde Scheele, Johanna Schönrock-Adema, A. Debbie C. Jaarsma, Janke Cohen-Schotanus

**Affiliations:** 1 Center for Education Development and Research in Health Professions (CEDAR), University of Groningen and University Medical Center Groningen, Antonius Deusinglaan 1, 9713 AV Groningen, The Netherlands; 2 St. Lucas Andreas Hospital (SLAZ), Amsterdam, The Netherlands

**Keywords:** Leadership development, Residency, Postgraduate medical education, Work-based learning

## Abstract

**Background:**

Within the current health care system, leadership is considered important for physicians. leadership is mostly self-taught, through observing and practicing. Does the practice environment offer residents enough opportunities to observe the supervisor leadership behaviours they have to learn? In the current study we investigate which leadership behaviours residents observe throughout their training, which behaviours supervisors report to display and whether residents and supervisors have a need for more formal training.

**Methods:**

We performed two questionnaire studies. Study 1: Residents (*n* = 117) answered questions about the extent to which they observed four basic and observable Situational Leadership behaviours in their supervisors. Study 2: Supervisors (*n* = 201) answered questions about the extent to which they perceived to display these Situational Leadership behaviours in medical practice. We asked both groups of participants whether they experienced a need for formal leadership training.

**Results:**

One-third of the residents did not observe the four basic Situational Leadership behaviours. The same pattern was found among starting, intermediate and experienced residents. Moreover, not all supervisors showed these 4 leadership behaviours. Both supervisors and residents expressed a need for formal leadership training.

**Conclusion:**

Both findings together suggest that current practice does not offer residents enough opportunities to acquire these leadership behaviours by solely observing their supervisors. Moreover, residents and supervisors both express a need for more formal leadership training. More explicit attention should be paid to leadership development, for example by providing formal leadership training for supervisors and residents.

## Background

Leadership is considered an essential competency for physicians, independent of whether they hold a formal leadership position [1–3]. Developing leadership skills is considered crucial to meet the challenges of future health care, which can be solved through leadership in managing and delivering high-quality, cost effective care ensuring patient safety. However, consensus on the importance of leadership competencies for medical practice does not guarantee explicit attention for leadership development in residency training programmes. The majority of universities and hospitals do not provide leadership training for students, residents and staff [4–6]. Consequently, what residents learn about leadership is self-taught. Their knowledge about leadership is mostly tacit and acquired through experience, for example by observing their supervisors’ behaviours and reflecting on situations encountered in practice [7, 8]. Does current medical practice itself offer enough possibilities to learn leadership by observation? To develop leadership in practice, it is important for residents that their supervisors display certain leadership behaviours, but it may be even more important that residents observe these leadership behaviours over the course of their training.

To investigate leadership in clinical practice, we used the Situational Leadership Theory (SLT), because this theory focuses on basic leadership behaviours that all physicians should demonstrate. The SLT is a transactional leadership theory that defines leadership as “the process of influencing the activities of an individual or group in efforts towards goal achievement” [9]. We tailored this leadership definition to our healthcare context and it now reads “the process in which supervisors’ leadership behaviours influence residents’ activities in efforts towards providing high quality care. In the SLT literature, two dimensions of leadership behaviours are defined: task-oriented and relation-oriented leadership behaviours [10]. Leaders who use task-oriented leadership behaviours make sure that their employees perform their task accurately and efficiently. Typical task-oriented behaviours are: planning work activities, clarifying objectives and monitoring employees’ performance and actions. Leaders who use relation-oriented leadership behaviours aim at maintaining good relationships with their employees. Typical relation-oriented behaviours are: showing support, showing appreciation for employees and two-way communication [11, 12]. Both kinds of behaviours are core issues of basic leadership [9] that should be learned by all physicians because they can support the performance of the medical team as a whole. Residents should experience these behaviours in medical practice in order to understand their usefulness and the relevance of learning them.

The most important premise of the SLT is that different situations require different leadership behaviours. The experience level of the employee is the most important determinant of which leadership behaviours to choose in a specific situation. According to this theory, adjusting leadership behaviours to the experience level of employees leads to better performance and well-being [9]. Translated to the clinical workplace, it is to be expected that supervisors use leadership behaviours that respond to the residents’ competence levels. The SLT states that a less experienced team member needs task-oriented leadership, which is instructive, and that a more experienced team member will benefit more from relation-oriented leadership in order to optimize his performance and well-being [9]. For residency, this would mean that an inexperienced, starting resident will observe task-oriented leadership behaviours more often, whereas an experienced, advanced resident will observe relation-oriented leadership behaviours more often. According to the SLT, residents should observe different leadership behaviours as they progress through residency. However, which leadership behaviours residents observe throughout their training is yet unknown.

Since all behaviours underlying the SLT are important for effective leadership, they should all be part of supervisors’ daily behaviour. For residents to be able to learn from observing their supervisors’ behaviour, it is very important that the latter display the whole range of situational leadership behaviours in practice. To shed some light on what actually happens in clinical practice, it is important to evaluate which leadership behaviours supervisors display in medical practice, according to their own perceptions.

According to literature, leadership training for students, residents and specialists is necessary to meet the demands of the current healthcare system [4, 6]. What do the stakeholders – residents and supervisors – think about leadership training? It is important to evaluate whether these stakeholders feel capable of being a leader and whether they feel a need for more formal leadership training, as is suggested in the literature.

In the current exploratory study, we investigated the extent to which residents observed different leadership behaviours of the SLT in medical practice and whether the observed behaviours changed in accordance with residents’ experience levels. We also investigated which leadership behaviours supervisors perceived to have displayed. In addition, we asked supervisors and residents if they felt capable as a leader and whether they experienced a need for formal leadership training.

## Methods

To answer our research questions, we performed two questionnaire studies. We asked two separate stakeholder populations of residency training, residents and supervisors, to complete a questionnaire about SLT leadership behaviours. Residents answered questions on leadership behaviours they observed in their supervisors and supervisors answered questions on the leadership behaviours they perceived to display themselves. Since the groups of residents and supervisors were approached separately, there was no information available about any possible connections between individual residents and supervisors.

### Ethical approval

Data were collected at a time when educational studies were exempt from Institutional Board Review in the Netherlands, under Dutch law. However, the data was gathered in accordance with established ethical standards and the Declaration of Helsinki [13–15]. Both residents and supervisors consented to participate, sent back their questionnaires voluntarily and anonymously.

### Study 1: residents

#### Context, participants and procedure

We attached our questionnaire on leadership to a larger survey on current professional issues and future perspectives of medical graduates of the University of Groningen in 2010. Residents who graduated our medical school between 2005 and 2010 participated in the study. They were asked to fill out the questionnaire that was sent to their home address, to put it in the anonymous return envelope that was provided in the package and send it to the first author.

#### Instrument

The extent to which residents observed task and relation-oriented leadership behaviours was measured with four items that had to be rated on a 4-point scale, ranging from 1 (never observed) to 4 (observed very often). All items were based on the Situational Leadership Theory of Hersey and Blanchard [9]. The leadership behaviours we measured are general behaviours that can easily be observed in many different situations. By deliberately not adding context, we tried to prevent that respondents would only refer to one particular situation or part of their work. We asked the residents to keep in mind the supervisor with whom they were working most of the time in clinical practice. Two items addressed task-oriented leadership: (1) “The supervisor tells exactly how, where, and when to perform tasks” (specific instructions), and (2) “The supervisor gives general directions on how to complete tasks” (global instructions). The other two items addressed relation-oriented leadership: (1) “There is two-way communication with the supervisor” (two-way communication), and (2) “The supervisor makes mutual decisions with the resident” (mutual decision-making).

In addition, residents were asked to indicate whether they felt capable as a leader and whether they had a need for formal leadership training (yes/no).

### Study 2: supervisors

#### Context, participants and procedure

We added our questionnaire on leadership to a larger longitudinal survey on current professional issues and future perspectives of medical graduates of the University of Groningen in 2010. Supervisors, who graduated our medical school in the 1970s and were currently working in training hospitals all over the Netherlands participated in this longitudinal study. They were asked to fill out the questionnaire on their supervisor leadership behaviours. The questionnaire was sent to their home addresses and they were asked to put the completed questionnaire in the anonymous return envelope that was provided in the package and send it to the first author.

#### Instrument

The extent to which supervisors reported to display task and relation-oriented leadership behaviours was measured using the same four items as described in Study 1. However, the statements were framed differently to enable the supervisors to rate items about their own leadership behaviours, The  answering scale ranged from 1 (never displayed) to 4 (very often displayed). The items addressing task-oriented leadership were framed as: (1) “I say exactly how, where and when to perform a task” (specific instructions) and (2) “I give general directions on how to complete a task” (global instructions). The items addressing relation-oriented leadership were framed as: (1) “There is two-way communication between me and the resident” (two-way communication), and (2) “I make mutual decisions with the resident” (mutual decision-making) [9].

In addition, supervisors were asked to indicate whether they felt capable as a leader and whether they experienced a need for formal leadership training (yes/no).

All participants provided informed consent. In accordance with the university’s privacy policy and Dutch law, the data were anonymized before analysis and handled with confidentiality.

### Analyses

To investigate the extent to which residents observed the SLT leadership behaviours and the extent to which supervisors’ said to display these behaviours, percentages were calculated per answer option of each of the four items. In the results section, we aggregated the data to summarize the results. A leadership behaviour that had been scored with 1 or 2 was considered as ‘not frequently observed’, and a behaviour with a score of 3 or 4 as ‘frequently observed.

To investigate whether supervisors adjusted their leadership behaviours in accordance with the residents’ competence levels, we first assigned each resident to one of three groups based on their years in training: starting (1–2 years in training), intermediate (3–4 years in training) and experienced (longer than 4 years in training). Subsequently, for each group we calculated percentages per answer option of the four items. We also calculated percentages to assess residents’ and supervisors’ perceptions of their own capability as a leader and their need for formal leadership training.

## Results

Residents (*N* = 117, response rate 82 %) observed *global instructions* and *two-way communication* most often (86 and 73 %, respectively) and *specific instructions* and *mutual decision-making* least often (64 and 59 %, respectively) in their supervisors’ leadership behaviour. More than one-third of the residents did not report having received any *specific instructions* on how to complete tasks and/or having experienced *mutual decision-making* in practice (Table 1).

**Table 1:** SLT (Situational Leadership Theory) behaviours observed by residents (*N* = 117)

Percentage (%) per answering option (1 = never observed, 4 = observed very often)				
SLT behaviour	1	2	3	4
Specific instructions	6	30	48	16
Global instructions	2	12	52	34
Two-way communication	8	19	36	37
Mutual decision making	8	32	44	15

The extent to which residents observed the four leadership behaviours did not structurally differ between the years of residency training. No pattern could be detected (Table 2).

**Table 2:** SLT behaviours observed by resident per experience level

Percentage (%) per answering option (1 = never observed, 4 = observed very often)				
Specific instructions	1	2	3	4
Starting level (*n* = 52)	0	29	55	16
Intermediate level (*n* = 36)	13	28	46	13
Experienced level (*n* = 29)	0	39	39	22
Global instructions				
Starting level	0	14	64	22
Intermediate level	2	15	57	26
Experienced level	6	0	71	24
Two-way communication				
Starting level	14	14	23	49
Intermediate level	4	19	47	30
Experienced level	8	19	36	37
Mutual decision making				
Starting level	8	36	39	17
Intermediate level	9	26	50	15
Experienced level	8	32	44	15

Supervisors (*N* = 201, response rate: 65 %) reported to display *two-way communication* most often, followed by *global instructions* (96 and 91 %, respectively). They reported to display *mutual decision-making* and *specific instructions* least often (80 and 57 %, respectively) (Table 3).

**Table 3:** SLT behaviours reported by supervisor (*N* = 201)

Percentages (%) per answering option (1 = never performed, 4 = performed very often)				
SLT behaviours	1	2	3	4
Specific instructions	12	31	49	8
Global instructions	1	7	54	37
Two-way communication	1	2	30	66
Mutual decision making	3	17	59	21

Both residents and supervisors perceived themselves as being capable leaders (83 and 73 %, respectively). Furthermore, 91 % of the residents and 49 % of the supervisors expressed a need for formal leadership training.

## Discussion and conclusions

One-third of the residents did not observe the leadership behaviours *specific instructions* and *mutual decision-making* during their training, irrespective of their competence level. Moreover, one-third or more of the supervisors reported not to display these leadership behaviours. Even though most residents and supervisors felt capable as a leader, the majority of residents and supervisors expressed a need for formal leadership training.

The fact that *specific instructions* and *mutual decision-making* had neither been observed by residents nor been reported to be displayed by supervisors suggests that residents cannot develop these leadership behaviours by merely observing what is happening in practice. Observing others and reflecting on observations are very important aspects of developing leadership behaviours [8]. Residents who do not observe these two leadership behaviours in medical practice may not be aware of these behaviours and how and when to apply them in order to improve their team’s performance and well-being. Possibly, they will never show or explore these behaviours themselves.

It may also be that *specific instructions* and *mutual decision-making* are neither observed by residents nor performed by one-third of the supervisors, because these behaviours do not fit residency training. Residents should be able to function independently with minimal supervision, which may imply that both behaviours are not appropriate. Both behaviours are very detailed and time-consuming on the part of the supervisor. However, it is also imaginable that these leadership behaviours benefit residents’ performance and confidence. Specific instructions, for example, will support residents’ performance of new routines and make them feel confident. Mutual decision-making, for example, may be empowering [16]. Further research is needed to establish the effects of not observing and performing these specific SLT behaviours for medical practice and, more specifically, for residency training. The perceived absence of these leadership behaviours raises some other questions as well. Why do supervisors not show certain leadership behaviours and how is the absence of certain behaviours influenced by residents’ development levels and context? In a broader sense, it raises the question which behaviours are or are not appropriate for the context of resident training.

Residents observed the same leadership behaviours, irrespective of their year of training, which suggests that supervisors’ leadership is not adjusted to the residents’ competence levels. According to the SLT, not adjusting leadership to competence levels will negatively influence residents’ performance and well-being [9]. Starting residents will benefit more from task-oriented leadership, whereas residents who progress through residency will benefit more from relation-oriented leadership. Possibly, such a shift from task to relation-oriented leadership is not necessary. Recent literature suggests that both task and relation-oriented leadership could be beneficial throughout residency, independent of residents’ competence levels. Residents often feel uncertain about their roles, because they are both physicians and trainees [17, 18]. It seems important that residents receive sufficient guidance to complete their tasks successfully. An instructive and directive approach – task-oriented leadership – could increase their trust and confidence and improve their performance. Social support and good communication – relation-oriented leadership – on the other hand might buffer against stress several residents experience with their new tasks and responsibilities [17–20]. Further research is needed to find out whether indeed both task and relation-oriented leadership behaviours benefit residents’ performance and well-being, independent of their competence level.

Residents and supervisors feel capable as leaders but both groups express a need for formal leadership training, which is in line with studies reporting that physicians experience a need for training in leadership competencies [21–23]. A first step to facilitate residents’ leadership development could be to make leadership behaviours more explicit in practice, as has been proposed for undergraduate medical education [5, 24]. Residents and supervisors should be made more aware of the importance of leadership and of how the working environment can be influenced by leadership. Explicit feedback on leadership competencies and assessment of leadership might help residents further develop their leadership competencies. Furthermore, from our study we learned that medical practice may not provide residents a chance to observe the whole range of basic SLT leadership behaviours which they should learn. Additional training in such behaviours may be beneficial. An example of how leadership training for residents could be tailored to practice is to deliberately practice leadership behaviours on the work floor and to discuss experiences in a structured manner in a peer group setting [5, 25]. In addition, supervisors could benefit from more formal leadership training, like workshops and leadership training programmes that could make them aware of the importance of certain leadership behaviours and how they could influence residents’ performance and well-being [26].

The Situational Leadership Theory [9] provides a useful framework for research on leadership behaviour in postgraduate medical education. The behaviours included in this theory can be considered “basic” leadership behaviours every physician needs to learn and which can easily be observed in medical practice. Since the residents’ supervisors are simultaneously superordinates and teachers, our study may raise questions about where leadership behaviours begin and teaching behaviours end. However, as we focused on supervisor leadership behaviours originating from the Situational Leadership theory, which strictly constructs general leadership behaviours and not includes teaching behaviours, we solely evaluated how supervisors and residents interacted in a working relationship.

Even though the SLT provided a useful framework for the current study, other leadership behaviours may also be important for medical practice, for instance, behaviours underlying the transformational theories [27]. These theories define leadership as inspiring employees and creating a vision for future innovations. To date, change agents become more important in medicine because the changing needs of the health care system have to be met. Typical change agents’ behaviours are transformational leadership behaviours. For residents to become change agents, it would be useful to observe transformational behaviours in practice. We limited our study to self-reported data about specific leadership behaviours from the perspectives of residents and supervisors. It could be argued that personal observations do not always comply with reality, because people tend to overestimate their abilities and give socially desirable answers [28] and, moreover, that observations by an independent researcher are more objective. However, to be able to develop leadership in medical practice, residents need to actually observe the basic leadership behaviours in their supervisors. Observations of their supervisors’ leadership behaviours are a prerequisite for developing their own leadership in practice. Finally, we limited our study to two separate groups of respondents from training hospitals. Therefore, we were not able to match the leadership behaviours supervisors reported to display and those the residents observed. Besides, as we limited our study to residents in training hospitals, we did not include general practitioners or other physicians working outside the hospital setting. How leadership is addressed in these specific contexts may be a topic for future research.

Our exploratory, empirical study offers insight into the experiences of residents and supervisors with a number of Situational Leadership behaviours performed in medical practice. More explicit attention to leadership development is necessary to enhance the use of these leadership behaviours in medical practice.
